# *Traveler*, a New DD35E Family of *Tc1/Mariner* Transposons, Invaded Vertebrates Very Recently

**DOI:** 10.1093/gbe/evaa034

**Published:** 2020-02-18

**Authors:** Wencheng Zong, Bo Gao, Mohamed Diaby, Dan Shen, Saisai Wang, Yali Wang, Yatong Sang, Cai Chen, Xiaoyan Wang, Chengyi Song

**Affiliations:** College of Animal Science & Technology, Yangzhou University, Jiangsu, China

**Keywords:** *Tc1/mariner* transposons, *Traveler*, DD35E, horizontal transfer, evolution

## Abstract

The discovery of new members of the *Tc1/mariner* superfamily of transposons is expected based on the increasing availability of genome sequencing data. Here, we identified a new DD35E family termed *Traveler* (*TR*). Phylogenetic analyses of its DDE domain and full-length transposase showed that, although *TR* formed a monophyletic clade, it exhibited the highest sequence identity and closest phylogenetic relationship with DD34E/*Tc1*. This family displayed a very restricted taxonomic distribution in the animal kingdom and was only detected in ray-finned fish, anura, and squamata, including 91 vertebrate species. The structural organization of *TR*s was highly conserved across different classes of animals. Most intact *TR* transposons had a length of ∼1.5 kb (range 1,072–2,191 bp) and harbored a single open reading frame encoding a transposase of ∼340 aa (range 304–350 aa) flanked by two short-terminal inverted repeats (13–68 bp). Several conserved motifs, including two helix-turn-helix motifs, a GRPR motif, a nuclear localization sequence, and a DDE domain, were also identified in *TR* transposases. This study also demonstrated the presence of horizontal transfer events of *TR*s in vertebrates, whereas the average sequence identities and the evolutionary dynamics of *TR* elements across species and clusters strongly indicated that the *TR* family invaded the vertebrate lineage very recently and that some of these elements may be currently active, combining the intact *TR* copies in multiple lineages of vertebrates. These data will contribute to the understanding of the evolutionary history of *Tc1/mariner* transposons and that of their hosts.

## Introduction

Transposable elements (TEs) are mobile DNA fragments in host genomes that are able to change their genetic environment and act as major factors that contribute to the evolution of genomes; they also play an important role in genomic structure and genetic innovation (Feschotte and Pritham 2007; Huang et al. 2012). TEs are distributed extensively in both eukaryotes and prokaryotes; however, they are far more abundant in eukaryotic genomes. Furthermore, in both prokaryotes and eukaryotes, there seems to be a direct positive correlation between genome size and TE abundance (Kidwell 2002; Touchon and Rocha 2007). TEs are major determinants of genome size (Hawkins et al. 2006; Gao et al. 2016). Forty-five per cent of the human genome consists of TEs (Lander et al. 2001) versus nearly 85% in the maize genome (Schnable et al. 2009). TEs are classified into two types (classes I and II) according to their mechanism of transposition. Class I elements (retrotransposons) are transposed via the reverse transcription of an RNA intermediate. Class II elements (DNA transposons) can be further divided into three major subclasses: the classical “cut-and-paste” DNA transposons, “rolling circle” DNA transposons, and “self-synthesizing” DNA transposons (Feschotte and Pritham 2007). Despite the differences in transposition mechanisms, some integrases of RNA TEs and transposases of DNA TEs are thought to have a common origin (Capy et al. 1997).

The *Tc1/mariner* superfamily is a “cut-and-paste” group of class II TEs that was first discovered in *Drosophila mauritiana* (*mariner*) (Jacobson et al. 1986) and *Caenorhabditis elegans* (transposon *C. elegans* number 1, *Tc1*) (Emmons et al. 1983) and is distributed extensively in eukaryotes (Haymer and Marsh 1985; Jacobson et al. 1986). The *Tc1/mariner* transposons generally have a size of 1,300–2,400 bp and encode a 340 amino acid (aa) transposase that is flanked by two terminal inverted repeats (TIRs) and dinucleotide target site duplications (TSDs) of TA (Lohe et al. 1996). Diverse families of this superfamily, such as DD34E*/Tc1*, DD34D*/mariner*, DD36E*/IC*, DD37D/*maT*, DD37E/*TRT*, DD39D, DD41D, DD×D*/pogo*, and DD×E, have been defined based on the phylogeny of the DDE conserved catalytic motif (Shao and Tu 2001; Bouuaert et al. 2015; Sang et al. 2019). DD34E/*Tc1* (Vos et al. 1993; Radice et al. 1994; Lam et al. 1996; Ivics et al. 1997; Sinzelle et al. 2005), DD×D/*pogo* (Tudor et al. 1992), and DD34D*/mariner* (Robertson 1993; Plasterk et al. 1999; Arkhipova and Meselson 2005; Nguyen et al. 2014) have been known for a long time and have been studied extensively, whereas DD37D/*maT* (Robertson and Asplund 1996; Gilchrist et al. 2014), DD39D (Jarvik and Lark 1998; Tarchini et al. 2000), and DD41D (Gomulski et al. 2001) were identified recently, with few reports being available; however, their evolution profiles, including taxonomic distribution, intrafamily diversity, and evolutionary dynamics in genomes are poorly understood. In contrast, DD36E/*IC* (Sang et al. 2019) and DD37E/*TRT* (Zhang et al. 2016) are newly discovered families with well-defined evolution profiles. DD37E/*TRT* was confirmed as a new subfamily within the *Tc1/mariner* superfamily and is present in bony fishes, the clawed frog, snakes, protozoans, and fungi; this widespread distribution of DD37E/*TRT* among fishes, frogs, and snakes is the result of multiple independent horizontal transfer (HT) events. DD36E/*Incomer* represents a unique DD36E motif that seems to have originated from DD34E and is mainly distributed across vertebrates (including jawless fish, ray-finned fish, frogs, and bats), with a restricted distribution in invertebrates (four species in Insecta and nine in Arachnida). HT events of DD36E/*IC* were also detected in vertebrates (Sang et al. 2019). In addition, new monophyletic clades of DD34E (termed *Gambol*) (Coy and Tu 2005) and DD37E (termed *Tnp*) (Puzakov et al. 2018) were identified that are distinct from the previously discovered DD34E/*Tc1* and DD37E/*TRT* families and form separate branches.

**Fig. 1.**
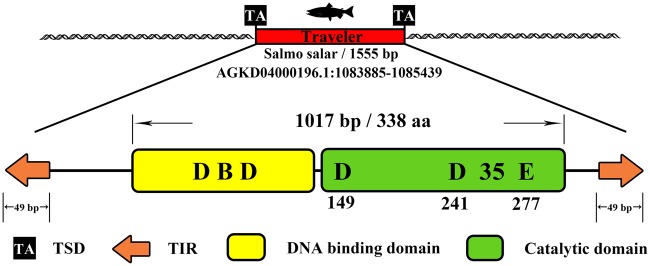
—Structural and functional components of representative *TR* elements in *Salmo salar*. Top, schematic representation of the transposon as a red rectangle with the length and the genomic coordinate of the representative *TR* element. The element contained a single gene encoding the transposase. The black squares represent TA TSD nucleotide sites, the orange arrows represent TIRs, the yellow rectangle represents the DNA-binding domain, and the green rectangle represents the catalytic domain.

The *Traveler* (*TR*) elements were first discovered in the *Salmo salar* genome via a TBlastN search using the *sleeping beauty* (*SB*) transposase (Ivics et al. 1997), which is a well-known DNA transposon of the *Tc1/mariner* superfamily. The intact *TR* element in *S.* *salar* has the typical structural organization of *Tc1/mariner* transposons, with TIRs flanking the segments of the transposon (∼1.5 kb) and transposase (338 aa); however, it comprises a unique DD35E motif (fig. 1) that differs from the typical DDE motif (DD34E) of the *Tc1* family (Lam et al. 1996), indicating that *TR* is a potential new family of *Tc1/mariner* transposons. To illustrate the evolution profiles of *TR* in genomes, we investigated the taxonomic distribution, structural organization, phylogenetic nature, and amplification dynamics of *TR*s. Our data revealed that *TR* is a new family that evolved recently from DD34E/*Tc1* and exhibits a restricted taxonomic distribution in vertebrates and recent invasion events in most detected lineages. Our study also identified multiple HTs of *TR*s in vertebrates. Overall, we discovered a unique DD35E transposon family, which expanded the diversity of the *Tc1/mariner* superfamily, thus promoting the understanding of the evolution of DNA transposons and their impact on animal genomes.

## Materials and Methods

### Retrieval of *TR* Elements

To assess the distribution of *TR* elements in genomes, the *TR* transposase sequence of *S.* *salar* was used to search the whole-genome shotgun contig database at the NCBI using TBlastN with a value of 1e^−100^. This transposon was manually determined to exist in a species when the catalytic domain (DD35E) of *TR* was detected. Significant hits were extracted with 1,000-bp flanking sequences, which were aligned to determine their boundaries. Subsequently, the representative sequence or consensus sequence of *TR* was searched against its host genome, to estimate copy number. All hits obtained that were >1,000 bp in size and had 80% identity were used to calculate the copy number. The consensus sequence of *TR* was reconstructed. In addition, transposons with a low copy number in the genome, which may be false-positive hits resulting from sequence contamination in the assembled genome or WGS, were verified further by mapping the flanking sequences of the transposon insertion to the host genome or to the genomes of closely related species; the unmapped transposons were designated as sequence contamination and were excluded from the analysis.

### Sequence Analysis and Phylogenetic Inference

Protein secondary structure predictions were performed using the PSIPRED program (http://bioinf.cs.ucl.ac.uk/psipred/) (McGuffin et al. 2000). Putative nuclear localization signal (NLS) motifs were predicted using PSORT (https://www.genscript.com/psort.html?src=leftbar). Multiple alignments were performed using the multiple alignment program ClustalW embedded in the BioEdit tool (Yang et al. 2003) and were manually edited and annotated using GeneDoc (Nicholas et al. 1997). The protein domains were identified using the profile hidden Markov Models with the online hmmscan web server (https://www.ebi.ac.uk/Tools/hmmer/search/hmmscan). TIRs were manually determined using the ClustalW program in the BioEdit tool. The consensus sequence was constructed using DAMBE (Xia 2018). A consensus sequence or representative sequence of each identified transposon was selected for further analysis in this study. The species divergence times were estimated using the online TimeTree program (http://www.timetree.org/) (Hedges et al. 2015). Sequence identities between the *TR* family and other families were measured via pairwise comparisons of full-length (FL) transposases using the BioEdit tool. The conserved DDE domains of the identified *TR* transposases and FL transposases were aligned to the representative TE families from the *Tc1/mariner* superfamily separately using MAFFT v. 7.310 (Yamada et al. 2016). The phylogenetic trees were inferred based on the conserved DDE domain (∼150 aa) (supplementary data set S1, Supplementary Material online) and the FL *Tc1/mariner* transposases (supplementary data set S2, Supplementary Material online) using the maximum likelihood method with the IQ-TREE program (Nguyen et al. 2015). The best-suited aa substitution model for these data was the VT+I+G4 model, according to BIC, which was selected by ModelFinder (Kalyaanamoorthy et al. 2017). The reliability of the maximum likelihood trees was estimated using the ultrafast bootstrap approach with 1,000 replicates.

### Pairwise Distances between the *TR* and *RAG1* Sequences

Pairwise distances between the different animal species included in this study were calculated for the *TR* and *RAG1* coding sequences, to test the HT hypothesis. Their accession numbers are listed in supplementary table S1, Supplementary Material online. Species for which we were unable to find the complete CDS region of the *RAG1* gene in the NCBI database were excluded from the analysis. Multiple alignments of *RAG1* (supplementary data set S3, Supplementary Material online) and *TR* (supplementary data set S4, Supplementary Material online) were generated using the MUSCLE program embedded in MEGA (v. 7.2.06) and were used to calculate the pairwise distances using MEGA (v. 7.2.06) (pairwise deletion, maximum composite likelihood) (Kumar et al. 2016).

### Evolutionary Dynamics Analysis

To compare *TR* dynamics among these species, the Kimura two-parameter distance was calculated using the calcDivergenceFromAlign.pl package from RepeatMasker (Tarailo-Graovac and Chen 2009).

## Results

### Narrow Taxonomic Expansion of *TR* Transposons

**Fig. 2.**
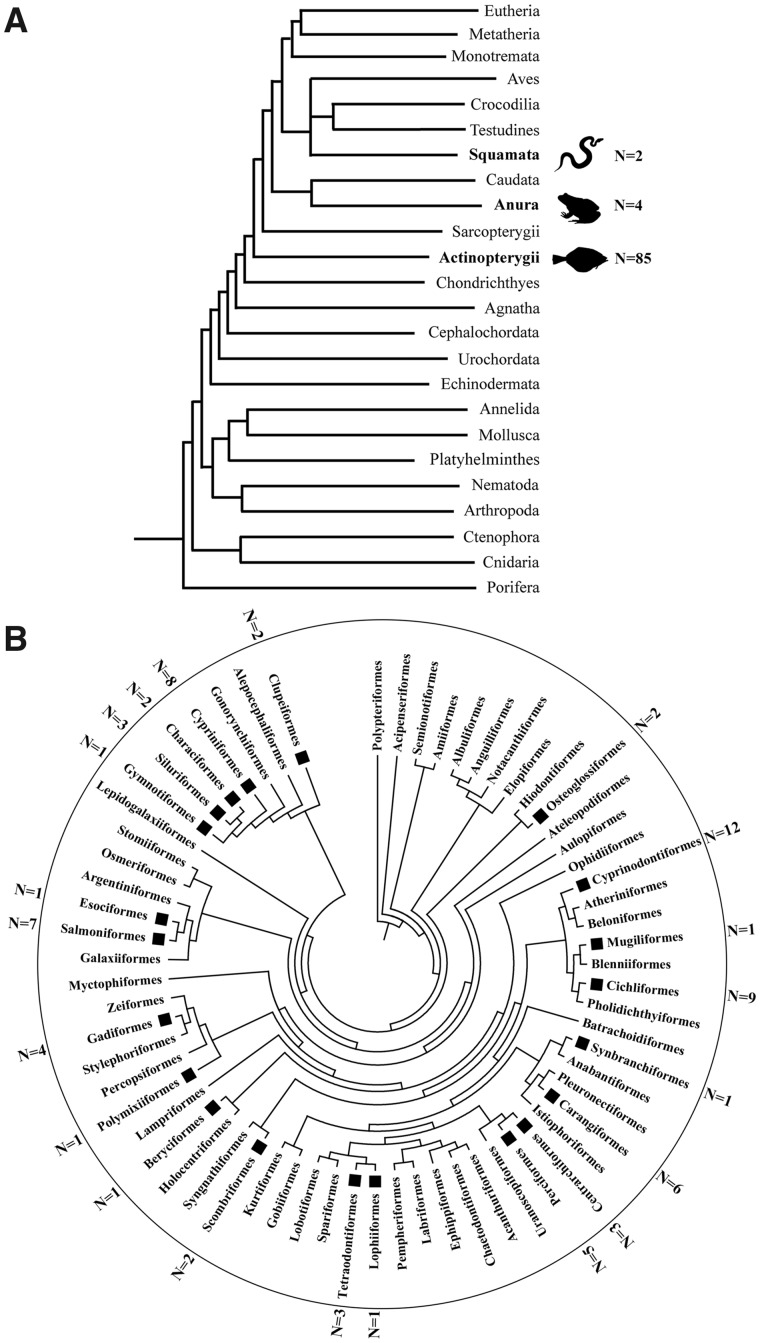
—Taxonomic distribution of *TR*s. (*A*) Taxonomic distribution of *TR* elements in the animal kingdom. The numbers next to the animal silhouettes represent the number of *TR*s detected in the species of each lineage. (*B*) Taxonomic distribution of *TR* elements in Actinopterygii. The taxonomic tree represents the distribution of the species identified in Actinopterygii (ray-finned fish) in their respective orders. The *TR*-positive orders are labeled with a square node and the number of *TR*-positive species is shown around the circle. The phylogenetic relationships were taken from the TimeTree database (http://timetree.org/) (Hedges et al. 2015).

To determine the taxonomic distribution of *TR*, the *S.* *salar TR* transposase sequence was used as a query to perform a TBlastN search in the NCBI whole-genome shotgun database, which contains all of the sequenced genomes from prokaryotes and eukaryotes. In turn, the newly obtained *TR* transposases were used as queries to identify additional *TR* elements. The TBlastN search revealed that this family has a very restricted taxonomic distribution in genomes because it was only present in one superclass (ray-finned fish) and two orders (anura and squamata) of vertebrates. In greater detail, this family exhibited a patchy distribution in vertebrates and was only detected in 85 species of ray-finned fish, 4 species of anura, and 2 species of squamata (table 1). Within the lineage of ray-finned fish, it invaded into 75 species of 22 defined orders and 10 unranked species (table 1 and supplementary table S2, Supplementary Material online). *TR* also invaded into four amphibian species (*Nanorana parkeri*, *Rana catesbeiana*, *Rhinella marina*, and *Xenopus tropicalis*) and two species of reptiles (*Python bivittatus* and *Salvator merianae*; fig. 2 and table 1). In invertebrates, a similarity with *TR* was detected in a single flatworm species (*Gyrodactylus salaris*), in which it encoded a truncated transposase. However, the flanking genomic sequences and the hallmarks (TIRs and TSDs) of *Tc1/mariner* were undetectable; furthermore, it was identified in a very short contig. Thus, this result seems to have stemmed from sequence contamination and was excluded from further analysis.

**Table 1:** Taxonomic Distribution of *TR*s

Taxonomic Distribution	Number of Species Containing a *TR*	Number of Species Containing an FL *TR*	Number of Species Containing an Intact *TR*	Length of the FL *TR*	Length of the Intact *TR*	Transposase Length of the Intact *TR*	TIR Length of the Intact *TR*	Number of Copies of the Intact *TR*	TSD
Actinopterygii	85	73	30	1,072–2,765	1,072–2,191	303–350	13–68	1–142	TA
Beryciformes	1	1	**—**	1,275	**—**	**—**	**—**	**—**	TA
Carangiformes	6	6	3	1,379–1,566	1,558–1,566	349–350	38–39	1–3	TA
Centrarchiformes	3	3	3	1,072–2,191	1,072–2,191	311–338	13–52	1–1	TA
Characiformes	2	2	**—**	1,505–1,556	**—**	**—**	**—**	**—**	TA
Cichliformes	9	6	**—**	1,520–2,047	**—**	**—**	**—**	**—**	TA
Clupeiformes	2	1	1	1,559	1,559	337	65	1	TA
Cypriniformes	8	8	4	1,535–1,557	1,554–1,557	349–349	18–38	3–142	TA
Cyprinodontiformes	12	12	7	1,228–1,579	1,461–1,579	303–338	16–28	1–37	TA
Esociformes	1	1	**—**	1,204	**—**	**—**	**—**	**—**	TA
Gadiformes	4	4	**—**	1,202–1,554	**—**	**—**	**—**	**—**	TA
Gymnotiformes	1	1	**—**	1,510	**—**	**—**	**—**	**—**	TA
Lophiiformes	1	1	**—**	1,534	**—**	**—**	**—**	**—**	TA
Mugiliformes	1	1	1	1,552	1,552	338	43	1	TA
Osteoglossiformes	2	2	1	1,559–1,565	1,559	305	38	1	TA
Perciformes	5	3	1	1,217–1,566	1,566	338	26	9	TA
Polymixiiformes	1	1	**—**	2,531	**—**	**—**	**—**	**—**	TA
Salmoniformes	7	7	5	1,548–1,567	1,548–1,567	319–338	30–68	1–110	TA
Scombriformes	2	1	**—**	1,565	**—**	**—**	**—**	**—**	TA
Siluriformes	3	3	2	1,195–1,553	1,527–1,553	324–349	26–38	1–8	TA
Synbranchiformes	1	1	**—**	1,580	**—**	**—**	**—**	**—**	TA
Tetraodontiformes	3	2	**—**	1,125–1,447	**—**	**—**	**—**	**—**	TA
Unrank	10	6	2	1,525–2,765	1,558–1,566	338–338	31–50	2–2	TA
Anura	4	4	2	1,545–1,944	1,563–1,565	338–350	26–39	2–>300	TA
Squamata	2	1	—	1,521	—	—	—	—	TA

We also found that most *TR* transposons were truncated: in ray-finned fish, more than half of the species (73/85) contained FL *TR* elements that comprised two detectable TIRs and TSDs; however, only 30 of them contained intact *TR* copies. Two species of frogs also contained intact copies of *TR*s (supplementary table S2, Supplementary Material online). The number of *TR* copies per genome varied significantly across species, ranging from one to several thousands (>80% of identity and >1,000 bp in length) in some ray-finned fish species, such as *Oncorhynchus kisutch*, *Oncorhynchus mykiss*, *Oncorhynchus tshawytscha*, *S.* *salar*, *Salvelinus alpinus*, and *Thymallus thymallus*, which exhibited 2,420, 3,843, 5,550, 3,259, 2,791, and 3,467 copies, respectively, which suggests that these transposons underwent species-specific proliferation in their host genomes. Among anura, >8,000 copies of *TR*s were detected in *Rhi.* *marina*, >2,000 of which were intact. The remaining three species of frogs, that is, *N.* *parkeri*, *R.* *catesbeiana*, and *X.* *tropicalis*, contained 59, 559, and 44 copies of *TR*s, respectively, but only *X.* *tropicalis* contained 2 intact copies of the *TR*. In addition, 255 copies of *TR*s were detected in one species of python (*P.* *bivittatus*) and 1 copy of *TR* was observed in one species of lizard (*Sal.* *merianae*). The single copy of *TR* detected in the snake was truncated but retained coding capacity for the transposase (344 aa); however, the TIR and TSD hallmarks were absent. Moreover, all FL *TR*s detected in lizards were truncated and exhibited loss of coding capacity (supplementary table S2, Supplementary Material online).

### Highly Conserved Structural Organization of *TRs*

**Fig. 3.**
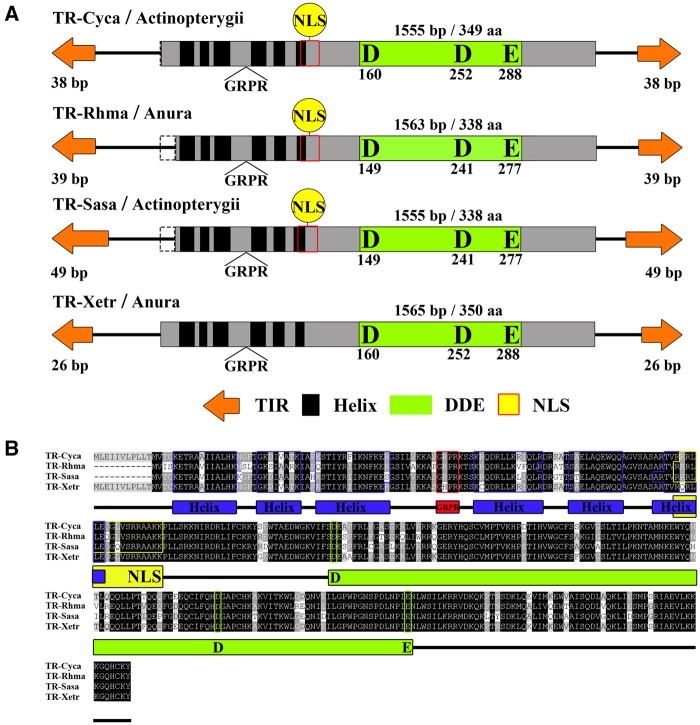
—Structural organization of *TR* transposons. (*A*) Structural organization of *TR* transposons. The orange arrows represent TIRs, the black rectangles represent HTH motifs, the black triangle represents GRPR sequences, the yellow circle represents the NLS, the green rectangles represent catalytic domains, and the gray region represents transposases. The dotted box represents the portion of the transposases that may be deleted in a particular species. (*B*) Alignment of the domains of *TR* transposases. We selected four representative species, that is, two ray-finned fish and two frogs. For species abbreviations, refer to supplementary table S2, Supplementary Material online.

The structural organization of *TR*s was highly conserved across different classes of animals, including fish, frogs, a python, and a lizard. Most of the intact *TR* transposons had a total length of ∼1.5 kb (range 1.0–2.2 kb) and harbored a single open reading frame encoding a transposase of ∼340 aa (range 304–350 aa) (fig. 3*A*). The length variations of transposons are caused by the variable length of the 5′- and 3′-untranslated regions. Several conserved motifs that are characteristic of *Tc1/mariner* transposons (Plasterk et al. 1999) were also observed in the *TR* transposase sequence. First, two helix-turn-helix (HTH) motifs were detected at the N-terminal of the transposase and may play a role in DNA binding (Nagy et al. 2004; Rousseau et al. 2004; Feschotte and Pritham 2007); each of these motifs consisted of three alpha-helices. Second, a GRPR motif was detected between the two HTH motifs. Third, an NLS was identified in most transposases that overlapped with the C-terminal of the second HTH motif. Finally, a catalytic triad DDE motif was observed within the catalytic domain, represented by 35 aa located between the second aspartic acid (D) and the glutamic acid (E) (fig. 3*B*). In addition, all *TR* elements identified here had short TIRs (13–68 bp) and contained a highly conserved CAGTC (51/78) or CAGCC (23/78) motif at the end of these repeats, which was flanked by canonical 5′-TA-3′ TSDs (supplementary table S2, Supplementary Material online). This differed from the motifs observed in several known DD34E/*Tc1* transposons, such as CAGTT in *SB* (Ivics et al. 1997), CAGTG in *Frog Prince* (Csaba et al. 2003), and CAGTG in *Passport* (Clark et al. 2009).

### Evidence of the Presence of HTs of *TRs* and Origin of *TRs*

**Fig. 4.**
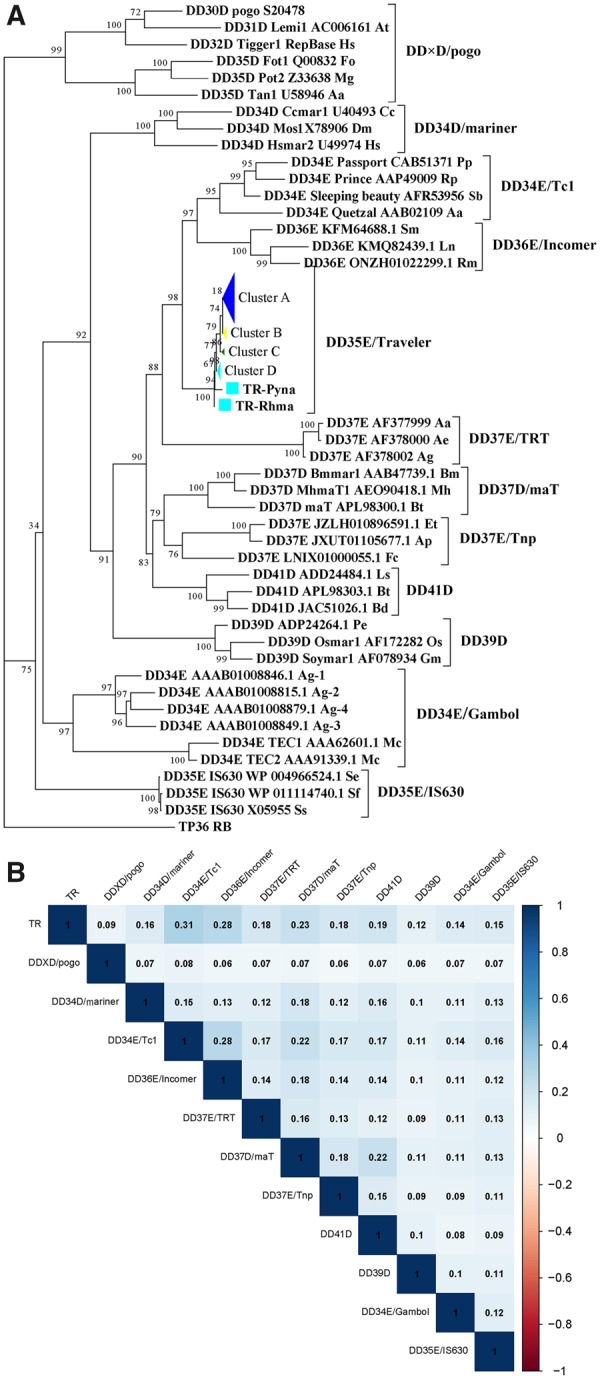
—Phylogenetic position of *TR* transposons relative to the families described previously. (*A*) Phylogenetic tree of *TR*s based on the alignment of the DDE domain. Bootstrapped (1,000 replicates) phylogenetic trees were inferred using the maximum likelihood method in IQ-TREE (Nguyen et al. 2015). The 11 known families of *Tc1*/*mariner* transposons (DD34E/*Tc1*, DD34D/*mariner*, DD36E/*Incomer*, DD37D/*maT*, DD37E/*TRT*, DD39D, DD41D, DD×D/*pogo*, DD34E/*Gambol*, DD37E/*Tnp*, and DD35E/*IS630*) were used as reference families (Coy and Tu 2005; Puzakov et al. 2018; Sang et al. 2019), whereas *TP36* was used as outgroup (Bao et al. 2009). For GenBank accession number and the abbreviated name of the host species of *Tc1/mariner* reference elements from other families, refer to supplementary table S3, Supplementary Material online. (*B*) Sequence identities between the *TR* family and eight other families. The sequence identities were measured by pairwise comparisons of FL transposases.

The phylogenetic position of *TR*s was inferred using the maximum likelihood method in the IQ-TREE based on the alignment of the conserved DDE domain (∼150 aa). The known families of *Tc1/mariner* transposons were used as reference families, and transposase 36 in *Rhodopirellula baltica* SH 1 (*TP36_RB*), which is an insertion sequence of bacteria that is close to the *Tc1/mariner* superfamily in phylogenetic position (Bao et al. 2009), was used as the outgroup. The access numbers of the reference *Tc1/mariner* elements are listed in supplementary table S3, Supplementary Material online. The phylogenetic tree showed that DD35E/*TR* formed a monophyletic clade and was defined as a new family (fig. 4*A* and supplementary fig. S1, Supplementary Material online); this family was more closely related to DD34E/*Tc1* and DD36E/*Tc1* than it was to other families of *Tc1/mariner*, which was confirmed by the phylogenetic tree that was generated using the FL transposases (supplementary fig. S2, Supplementary Material online). To confirm the evolutionary relationship between these families, we also generated a sequence identity matrix using the FL transposases. The matrix indicated that *TR*s exhibited the highest sequence identity to DD34E/*Tc1*, followed by DD36E/*IC* (fig. 4*B*). Therefore, we assumed that *TR* evolved from the DD34E/*Tc1* family independently from DD36E/*IC* transposons and formed a separate family within the *Tc1/mariner* superfamily.

**Fig. 5.**
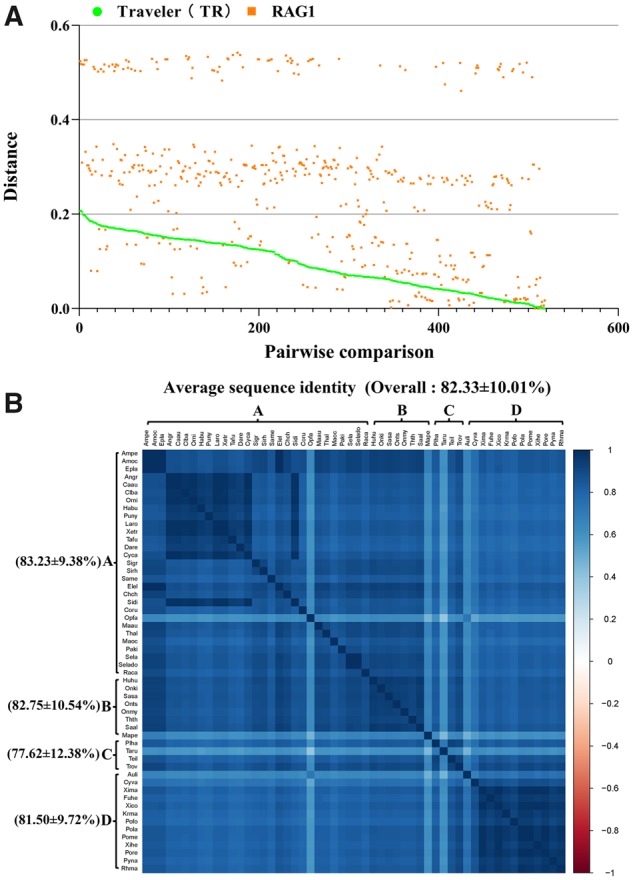
—HT analysis and sequence identities of *TR* transposons in vertebrates. (*A*) Graph illustrating the pairwise distances of *TR* and *RAG1* between the species included in this study. The distances were obtained from all possible pairwise comparisons (*n* = 518; labeled on the *x* axis) between the 29 (Cluster A), 8 (Cluster B), 4 (Cluster C), and 13 (Cluster D) species in which *TR*s were identified. (*B*) Sequence identities between *TR* elements among species. The sequence identities were measured by pairwise comparisons of FL *TR* consensus sequences or the representative sequence (for species abbreviations, refer to supplementary table S2, Supplementary Material online).

Based on the phylogenetic analysis, the *TR* elements were classified into four clusters (A–D): cluster A was detected in 26 ray-finned fish species, 2 anura species, and 2 squamata species; clusters B and C were present in 8 ray-finned fish species and 4 ray-finned fish species, respectively; and cluster D was detected in 12 ray-finned fish species and 1 anura species (supplementary fig. S1, Supplementary Material online). The observation that these four clusters, and even the whole *TR* family, exhibited a discontinuous distribution in animals ruled out the possibility that *TR* elements were vertically inherited from the last common ancestor of these species. To corroborate this conclusion, pairwise distances between the recombination-activating gene 1 (*RAG1*) and all consensus sequences or representative sequences of *TR*s were calculated and compared (supplementary table S4, Supplementary Material online). *RAG1* is an ideal locus for testing hypotheses about phylogeny and diversiﬁcation times in vertebrates (Hugall et al. 2007; Gilbert et al. 2012; Zhang et al. 2016). The distances of almost all pairwise comparisons (447/518) were extremely small (0.094 ± 0.055) compared with those calculated for *RAG1* (0.255 ± 0.154) (fig. 5*A*). Almost all *TR* pairwise distances computed here involved species that diverged from each other >212.8 Ma (supplementary table S4, Supplementary Material online). Given these large divergence times and the absence of purifying selection on *TR* sequences, the extremely low pairwise *TR* distances calculated here seem to be incompatible with a scenario that invokes vertical inheritance of these transposons from the ancestor.

Furthermore, in many cases, the sequence identities of *TR*s were extremely high compared with the divergence time of their hosts (fig. 5*B*). For example, >83.63% identity was observed between *TR*s in the fish and frog, which diverged >435 Ma. Similarly, the fish and lizard, which shared the last common ancestor ∼435 Ma, showed >80.14% identity (fig. 5*B*). This value is unexpectedly high considering the deep divergence detected between their hosts. The phylogenetic tree (supplementary fig. S1, Supplementary Material online) and TimeTree (supplementary fig. S3, Supplementary Material online) revealed incongruence between the *TR* and host phylogeny, which, in combination with the discontinuous distribution of *TR*s in animals, strongly suggests that *TR* elements might have been exposed to multiple events of HT.

### Evidence of Recent Invasions of *TRs* in Vertebrates

**Fig. 6.**
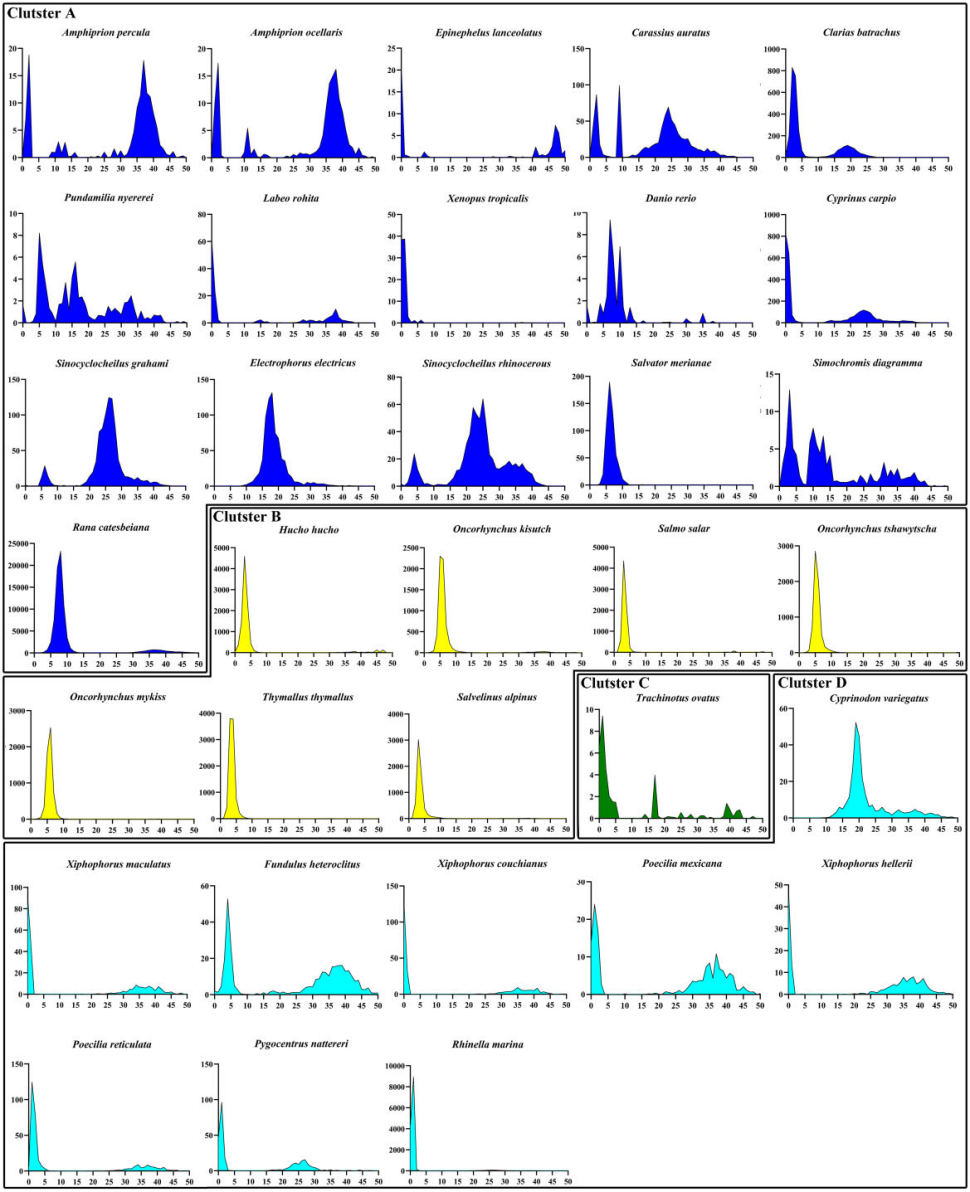
—Evolutionary dynamics of *TR*s in vertebrates. RepeatMasker utility scripts were used to calculate the K divergence from consensus sequences or the representative sequence (Tarailo-Graovac and Chen 2009). Species with less than ten copies of *TR*s in their genomes were excluded from the analysis. The *y* axis represents the coverage (kb) of each *TR* element in the genome and the *x* axis indicates the Kimura divergence estimate.

To illustrate further the evolution profiles of *TR* elements in vertebrates, we compared the evolutionary dynamics of *TR* elements across species and clusters using a Kimura divergence analysis and sequence identity, the results of which are summarized in figures 5*B* and 6. The sequence identity matrix showed that the overall average sequence identity (82.33 ± 10.01%) of *TR*s across species was substantially higher than that reported previously for DD36E/*IC* (52.48 ± 19.19%) (Sang et al. 2019). Each cluster, including clusters A (83.23 ± 9.38%), B (82.75 ± 10.54%), C (77.62 ± 12.38%), and D (81.50 ± 9.72%), displayed a high-sequence identity between species (fig. 5*B*), indicating that these four clusters may represent relatively recent HT events. The Kimura divergence estimations of *TR* elements revealed differential evolutionary dynamics of *TR*s in vertebrates because most species in cluster A experienced multiple waves of invasion of *TR*s, whereas most species in the remaining three clusters experienced a single wave amplification of *TR*s. Moreover, all species in clusters B, C, and D, with the exception of the TR in *Cyprinodon variegatus*, and some species in cluster A (*Amphiprion ocellaris*, *Amphiprion percula*, *Carassius auratus*, *Clarias batrachus*, *Cyprinus carpio*, *Epinephelus lanceolatus*, *Labeo rohita*, *Simochromis diagramma*, and *X.* *tropicalis*) exhibited very low Kimura divergences (<5%) (fig. 6), indicating that these species experienced very recent invasions of *TR*s. These data, combined with the discovery of intact *TR*s and high-sequence identity in these species, suggest that this family of transposons is a clade of *Tc1/mariner* that evolved very recently and may still be active in some lineages of animals.

## Discussion

### Reorganization of the DD34E*/Tc1* Family Based on Conserved Catalytic Motifs

DNA transposons, such as *piggyBac*, *P element*, *hAT*, and *Tc1/mariner*, usually transpose through a cut-and-paste mechanism. They are characterized by the presence of TIRs flanking a gene encoding a transposase that catalyzes the transposition reaction (Hickman and Dyda 2015). Despite the differences in transposition mechanisms, the transposases of some DNA elements are thought to have evolved from a common origin and share similar motifs in their catalytic domain (Capy et al. 1996). The *Tc1/mariner* superfamily is ubiquitous and forms the largest group of eukaryotic class II TEs. The common motif in these families is a conserved D (Asp) DE (Glu) or DDD catalytic triad, and multiple distinct intrafamilies have been identified to date based on this conserved catalytic domain (Bouuaert et al. 2015; Sang et al. 2019). This study provided the first in silico evidence of a new family (DD35E/*TR*) of this superfamily of transposons, which displayed the closest phylogenetic relationship and highest sequence identity to the DD34E/*Tc1* family, strongly indicating that it may have evolved from this family. This represents the second discovery of a sister family of DD34E/*Tc1* in animals, after the initial discovery of DD36E/*IC* in animals very recently, which also exhibited the closest phylogenetic relationship with the DD34E/*Tc1* family and was suggested to have originated from this family (Sang et al. 2019). Our previous study indicated that the DD34E/*Tc1* transposons display a high diversity at the family level because at least five distinct clusters or subfamilies (*Passport*-like, *SB*-like, *Frog Prince*-like, *Minos*-like, and *Bari*-like) were identified (Gao et al. 2017). In fact, the DD38E transposons identified in sturgeon have also been proposed to be a close sister family of DD34E/*Tc1* (Pujolar et al. 2013). These data suggest that the DD34E/*Tc1* transposons exhibit an unexpected diversity and may evolve into many families as a common ancestor. The systematic definition of the diversity of the DD34E/*Tc1* family in future studies may help illustrate the evolution landscapes of this family, as well as of the *Tc1/mariner* superfamily.

### Very Recent Invasions of *TRs* in Vertebrates

Several lines of evidence from the current study also supported the hypothesis that the DD35E/*TR* is a family that evolved very recently from the DD34E/*Tc1* transposons. First, most other families of *Tc1*/mariner, such as DD37E/*TRT* and DD36E/*IC*, seem to be distributed in both vertebrates and invertebrates, some of which are even very widely distributed, such as DD34E/*Tc1* (Vos et al. 1993; Radice et al. 1994; Lam et al. 1996; Ivics et al. 1997; Sinzelle et al. 2005) and DD34D/*mariner* (Robertson 1993; Plasterk et al. 1999; Arkhipova and Meselson 2005; Nguyen et al. 2014); in contrast, DD35E/*TR* seemed to exhibit the narrowest taxonomic distribution in animals and was only detected in vertebrates. Second, the average sequence identities of *TR*s between species across the four clades were very high (>80%), which differed from that observed in other families, such as DD37E/*TRT* (Zhang et al. 2016) and DD36E/*IC* (Sang et al. 2019), in which some clades exhibited high identity, whereas others displayed low-sequence identity (Sang et al. 2019). Third, the analysis of the evolutionary dynamics of *TR*s in these species revealed that most invasions were recent, with a Kimura divergence <5%, which was confirmed by the detection of many intact copies in these species. Taken together, these data indicate that *TR*s represent very recent invasion events in animals and may still be active in many species.

### HT Events of *TRs*

Horizontal transfer is the transmission of genetic material by means other than parent-to-offspring ones, which is a common occurrence in bacteria (Gogarten and Townsend 2005) but is considered a rare event in eukaryotes (Kidwell 1993; Andersson 2005). However, a growing body of evidence suggests that the HT of TEs, a particular type of HT, was very common during the evolution of eukaryotes; moreover, it is recognized increasingly as a source of genomic innovation (Wallau et al. 2012; Husnik and McCutcheon 2018). Multiple examples of HT events in vertebrates have been defined well, including diverse DNA transposon superfamilies, such as *hAT* (Gilbert et al. 2012), *PiggyBac* (Pagan et al. 2010), *Chapaev* (Zhang et al. 2014), and *Tc1/mariner* (Kuraku et al. 2012; Oliveira et al. 2012; Zhang et al. 2016), indicating that DNA transposons play important roles in shaping the evolution of genomes in vertebrates. In addition, our data revealed that the taxonomic distribution of *TR*s is limited, probably because of the young invasion history of this family in animals or the low promoter activity of *TR* transposases. Promoter strength has been suggested as a driving force of the transposon HT process (Palazzo et al. 2017). A blurry promoter, which is defined as a common feature of diverse *Tc1* and *mariner* elements, including *Bari*, *Sleeping Beauty*, and *Hsmar1*, may play roles in their evolutionary success and is probably involved in overcoming the barriers that exist between the transcriptional machinery of unrelated species (Palazzo et al. 2019). However, this feature is not found in the *hobo* transposon and LTR retrotransposons (Palazzo et al. 2019) and may be absent in *TR* elements, which constitutes a barrier to the HT of *TR*s. Although the mechanism of HT remains unclear, bacteria and pathogens may play a facilitating role, and parasites (such as viruses, ticks, nematodes, and insects), which engage in long-lasting and physical contact with their hosts, may act as shuttles or vectors for the HT of TEs between species (Wallau et al. 2018). The current study provided evidence of the presence of HT events of *TR* in vertebrates; however, the vectors of the HT events of this family remain unclear. Our data revealed that *TR* was mainly distributed in ray-finned fish, anura, and squamata, and no parasites of these species, which are potential vectors of HT of *TR*s in vertebrates, were detected. Although lampreys, which are opportunistic parasitic feeders that attach to large fish using their cup-like mouth to suck their blood and body fluids, were suggested as possible vectors of HT events of DNA transposons in ray-finned fish (Kuraku et al. 2012; Zhang et al. 2014), they were absent from the list of *TR* invasion species, which excluded their role as a vector of HT of *TR*s in ray-finned fish. In addition, a Blast search against the Nucleotide Collection (nr/nt) database at the NCBI that was aimed at identifying potential vectors using *TR* transposases as queries did not identify any *TR* homology sequences other than those detected previously. Thus, the potential transmission vectors of HTs of *TR* transposons remain unknown. 

## Supplementary Material

evaa034_Supplementary_Data
