# Supplementary material for: Traveler, a New DD35E Family of Tc1/Mariner Transposons, Invaded Vertebrates Very Recently
Source: Genome Biol Evol. 2020 Feb 18;12(3):66–76. doi: 10.1093/gbe/evaa034 (PMC7093834; doi:10.1093/gbe/evaa034)
Supplement: evaa034_Supplementary_Data [file evaa034_supplementary_data.zip › Supplementary figures S1-S3.docx]

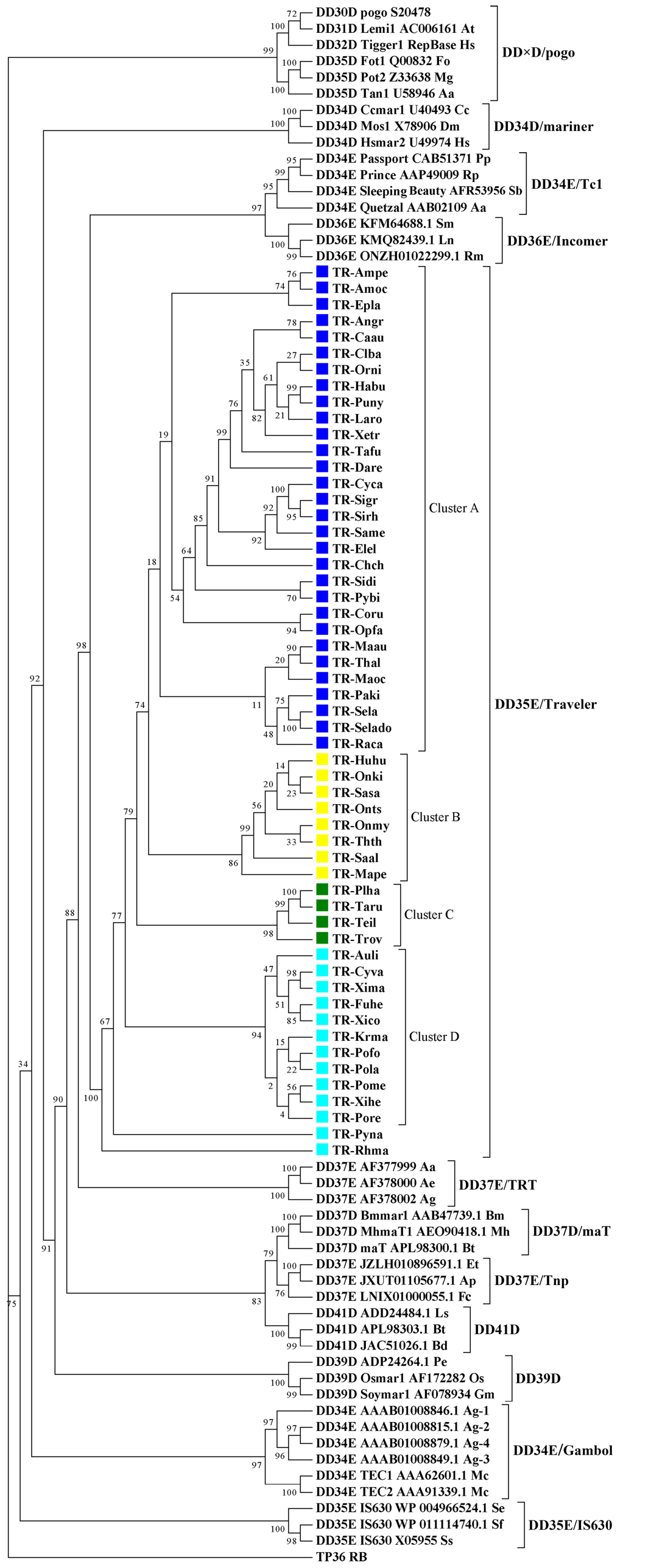


**Figure S1: Uncollapsed phylogenetic tree of *TR*s based on the alignment of the DDE domains.** Species with only highly fragmented copies and incomplete DD34E motifs in their genome were not included in this analysis. The phylogenetic tree was inferred using the maximum likelihood method with the IQ-Tree program. *TP36* transposase was used as an outgroup.


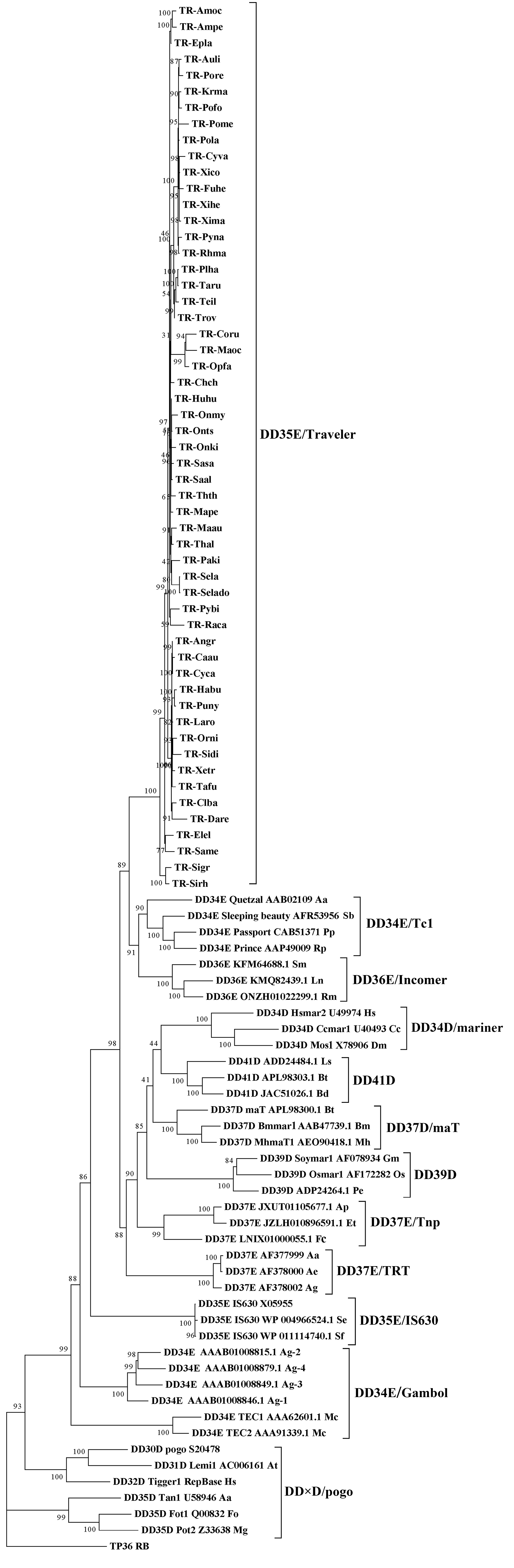


**Figure S2: Uncollapsed phylogenetic tree of TRs based on the alignment of the full-length transposases.** The phylogenetic tree was inferred using the maximum likelihood method with the IQ-Tree program. *TP36* transposase was used as an outgroup.


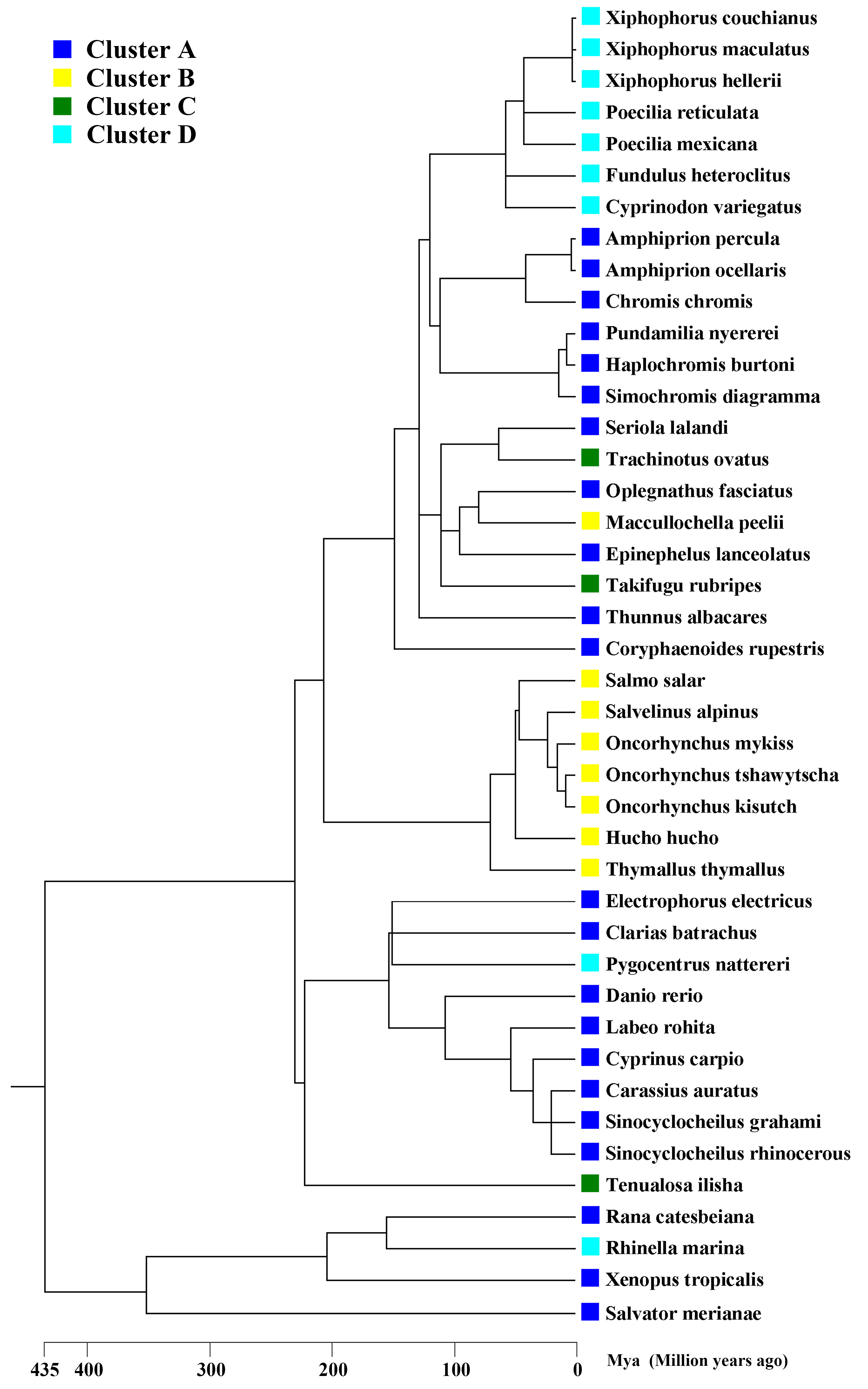


**Fig. S3** Time tree of species harboring *TR* elements identified in this study are obtained from the TimeTree database (<http://timetree.org/>).
